# Soil conditions modify species diversity effects on tree functional trait expression

**DOI:** 10.1038/s41598-024-67512-w

**Published:** 2024-07-24

**Authors:** Andréa Davrinche, Sylvia Haider

**Affiliations:** 1 Institute of Ecology, Leuphana University of Lüneburg, 21335 Lüneburg, Germany; 2 Institute of Biology, Martin Luther University Halle-Wittenberg, 06108 Halle, Germany; 3 German Centre for Integrative Biodiversity Research (iDiv) Halle-Jena-Leipzig, 04103 Leipzig, Germany; 4 Research Centre for Ecological Change (REC), Organismal and Evolutionary Biology Research Programme, University of Helsinki, 00014 Helsinki, Finland

**Keywords:** Complementarity, Controlled experiment, Functional traits, Microorganisms, Phosphorus fertilization, Soil nutrients, Spectroscopy, Within individual variation, Biodiversity, Plant ecology

## Abstract

Examples of positive effects of biodiversity on ecosystem functions have kept accumulating in the last two decades, and functional traits are considered suitable tools to explain their underlying mechanisms. However, traits are rarely studied at the scale where these mechanisms (e.g., complementarity) are likely to originate, that is, between two interacting individuals. In an 18-month greenhouse experiment, we investigated how species diversity (i.e., monospecific or heterospecific tree pairs) affects within-individual leaf traits expression and variation and how this effect is modified by soil conditions. While resource addition through phosphorus fertilization partly strengthened the diversity effects, inoculation of soil microbiota (potentially leading to increased resource accessibility) resulted in counter effects. Hence, in contrast to our expectations, we did not find synergistic effects of the two soil treatments, but we found distinct effects on species following an acquisitive or conservative growth strategy. Overall, our study showed that the effect of species diversity on young trees’ adaptability and resource-use strategy needs to be considered alongside soil biotic and abiotic aspects. The influence of soil conditions on species diversity effects is essential to understand mechanisms behind complementarity at the individual level, which ultimately translate to the community scale.

## Introduction

In the last decades, numerous studies have pointed out the prominent role of biodiversity as a main driver of ecosystem functioning and its associated services. Among the multitude of functions species-rich forests provide (i.e., multifunctionality^1^), the relationship probably most often studied is the effect of plant diversity on productivity^2–4^. For exploring the link between biodiversity and ecosystem functioning (BEF), inferring ecosystems’ responses from plant functional traits has emerged as an efficient approach to shed light on the mechanisms behind BEF relationships.

Some traits can stand directly as proxy for ecosystem functions, as for example plant height for estimating aboveground biomass and hence productivity, or specific leaf area (SLA) for photosynthetic capacity^5^. In addition, traits and their correlations also reflect plants’ ecological strategies. Aboveground, leaf traits are indicators of the plant species’ position within the leaf economics spectrum (LES)^6^, a gradient capturing species growth strategies from acquisitive to conservative resource use. At one end of the spectrum, species with an acquisitive growth strategy, characterised by a fast growth, invest resources into ‘cheap’ structures with a high turnover. At the other end, slow growing conservative species have a slow resource uptake and invest into costly, long-lasting structures. Typically, an acquisitive strategy translates into high values in leaf traits related to resource acquisition and use (e.g., SLA, leaf nitrogen, leaf cations) whereas a conservative strategy is reflected by leaf traits related to structural and physical defensive functions (e.g., leaf dry matter content, leaf carbon). While observed at a global scale in different growth forms^7^, the LES has also be found to be detectable in small sets of species^8^ or closely related species^9^, and has even been used to describe varying patterns within species (for example along environmental gradients^10–12^).

Depending on the position of the species within the LES, leaf traits can have more or less potential to vary. Indeed, species with a fast turnover (i.e., acquisitive species) have been found to build new leaves phenotypically adjusted to their local environment, which is less frequent for conservative species^13–15^. Hence, an acquisitive strategy is more likely to enable plants to keep pace with changing growing conditions.

Traits and their variation have received much attention at the species, and more recently the within-species level, but have rarely been investigated within plant individuals. Yet, the individual is the scale at which traits are defined^16^, and where local biotic interactions first occur before to shape higher scales’ processes. Interactions between plant individuals are known to be strongly driven by competition for resources^17,18^, as individuals share the needs for similar resources, a fortiori when they belong to the same species. Increased species diversity may therefore reduce the strength of competition among individuals, because of differences in resource needs and uptake between species, which might lead to complementarity in resource use^2,19^.

Moreover, species diversity can enhance soil biota diversity and activity, and hence support the nutrient cycle and the availability of resources in plant-usable forms. For example, in a long-term subtropical forest experiment, species diversity has been suggested to lead to an increase of available nutrients through higher microbial diversity^20^, higher litter abundance^21^ and faster litter decomposition^22,23^.

In addition to reducing competition, diversity has also been shown to modify the environment through positive effects of one species benefitting another (i.e., facilitation) for example by alleviating abiotic pressures (e.g., enabling a hydraulic lift increasing belowground water availability through different rooting lengths) or by influencing biotic variables (e.g., diluting species-specific soil pathogen loads) ^24^.

These positive effects of diversity, mitigating unfavourable environments and increasing resource availability, have translated into traits shifting towards more acquisitive values (i.e., a faster growth strategy) as opposed to more conservative values in non-diverse environments (i.e., a slower growth strategy)^25,26^. As for trait values, there is a considerable lack of information on the identity and importance of the drivers of trait variation at the individual scale, as well as whether they compare to patterns found at the species level^19,21^. The very few studies that address trait variation at this scale reported an increase of within-individual trait variation with diversity, as the higher resource availability allows for a wider range of trait values^27^, but trait variation was also observed to decrease in response to reduced inter-specific competition and a lesser need for variation^27,28^.

As described above, diversity can influence the availability of resources for individual trees and consequently affect functional traits and trait variation. Of course, soil properties may also directly act upon traits and ultimately ecosystem functions. This may happen through the resources themselves (that is, the amount of nutrients) but also their availability, resulting from interactions with soil biota. Indeed, higher nutrient amounts have been found to result in greater values for traits reflecting a more acquisitive growth strategy^29,30^ as well as trait variation^31^. Among the nutrients essential to plant growth, phosphorus in particular has been shown to be a major determinant for plant metabolism, and one of the most limiting nutrients for plant growth^32^. Indeed, phosphorus plays a role not only as a direct input in the mineral nutrition of the plant, but also in lifting co-limitations with other nutrients (for example with nitrogen)^33^. Soil micro-organisms may provide an improved access to and/or absorption of resources through positive plant-micro-organism relationships. Therefore, their presence may have comparable effects on plant traits compared to a direct input of plant-usable nutrients^34^. Indeed, soil microbiota has been found to enhance both availability and access of belowground resources for plants through higher mineralization rates^35^, to increase the nutrient pool (e.g., as a result of microbial feeding interactions^36^), and to facilitate nutrient transportation and absorption (e.g., in root cells colonised by symbiotic mycorrhiza^37^). In addition, microbial activity can protect the plant against soil pathogens, for example through the role of mycorrhiza^38^ or the production of bacterial antifungal metabolites^39^, thus indirectly favouring plant nutrient uptake and growth.

Although we can assume similar effects of more fertile soils and increased nutrient availability through plant species diversity on traits and their variation, the interplay of soil nutrients and plant species diversity has barely been addressed before. However, in the probably only existing field study on this interaction, the effect of soil nutrient availability on trait variation within individual trees was found to depend on the diversity of the neighbouring tree species. The results suggested that higher belowground resources could reduce competition, and hence decrease the need to vary for trees surrounded by a low diversity. Inversely, higher resource supply could increase variation for trees surrounded by higher diversity, as it enables the maximization of a tree’s adaptability to changing environmental conditions^27^.

**Figure 1:**
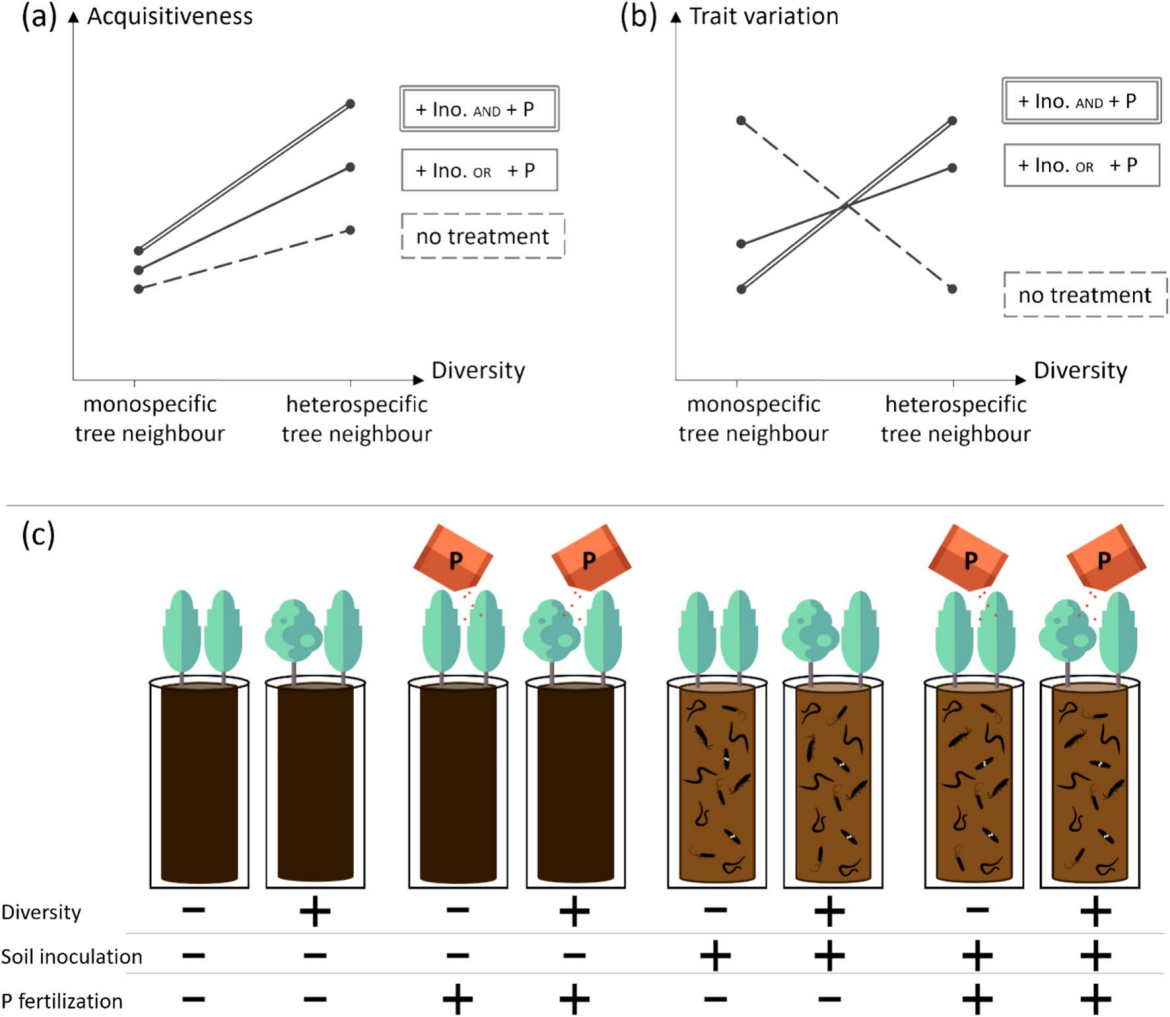
Expected trait acquisitiveness (**a**) and trait variation (**b**) of a focal tree in response to increasing species diversity (from mono to heterospecific tree neighbour; dashed line), with inoculation of the native soil microbiota (+ Ino; solid line), phosphorus fertilization (+ P; solid line), or both soil treatments together (+ Ino and + P; double line). (**c**) Experiment design. Trees are planted in mono- or heterospecific pairs (diversity) in soil either sterilized or inoculated with microbiota (soil inoculation) with addition or not of phosphorus (P fertilization).

In the 18-month experimental study under controlled conditions presented here, we aim at understanding the effect of tree species diversity on leaf functional traits and their variation. Focusing on trees with either a monospecific or a heterospecific neighbour, we propose to disentangle how soil nutrient availability (here, manipulated through phosphorus fertilization) and soil biota (through inoculation of the tree species’ native soil) modify diversity effects at the individual tree level. Therefore, we hypothesize that:

H1. The acquisitiveness of trees increases with tree species diversity, a higher amount of nutrients (phosphorus fertilization) and better access to nutrients (through soil microbiota added with soil inoculation). The nutrient-related factors’ effects are additive, and both enhance the effect of diversity (Fig. 1a).

H2. Trait variation decreases with species diversity, but this relationship is inversed with an increase in nutrients (phosphorus fertilization) or a better access to nutrients (through soil microbiota added with soil inoculation). Specifically, soil treatments independently, and a fortiori combined, result in lower variation for trees at low diversity and higher variation at higher diversity (Fig. 1b).

## Results

### Species growth strategy

Based on a principal component analysis, two species clusters were distinguished along the first axis which corresponded to the acquisitive-conservative spectrum (high SLA, leaf N, leaf P, and high LDMC, leaf C:N, respectively; Supp. Fig. S1). Driven by acquisitive-related traits, *Quercus serrata* (*Qs*), *Choerospondias axillaris* (*Ca*), *Sapium sebiferum* (*Ss*), *Koelreuteria bipinnata* (*Kb*), and *Quercus fabri* (*Qf*), were separated from more conservative *Cyclobalanopsis glauca* (*Cg*), *Schima superba* (*Ssu*), and *Rhus chinensis* (*Rc*) (Supp. Table S1).

### Leaves’ trait responses

**Figure 2:**
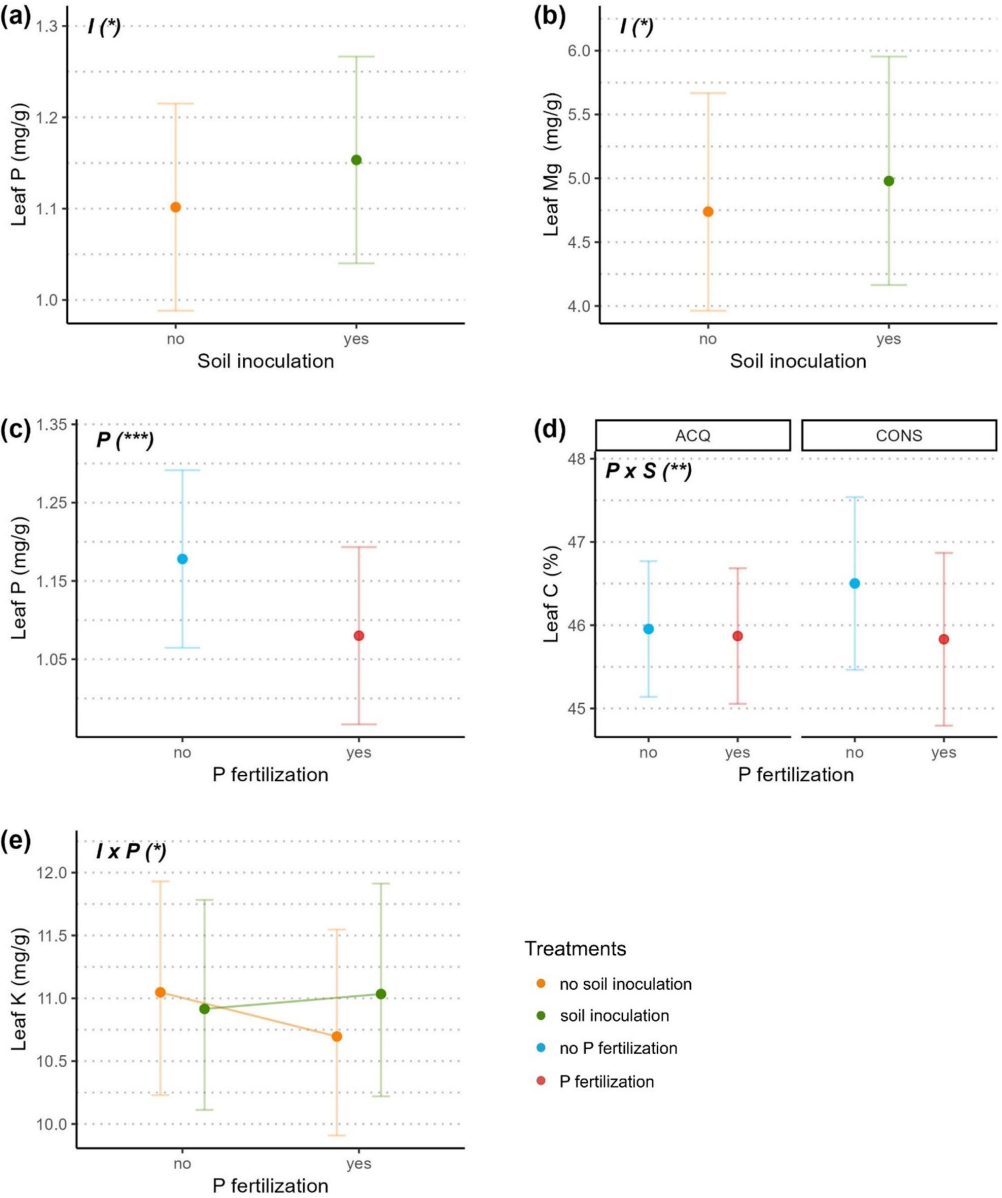
Effect of soil inoculation with microbiota (**a**, **b**), phosphorus fertilization (**c**; **d**) and the interaction of both soil treatments (**e**) on leaf traits. The effect of phosphorus fertilization on leaf C (**d**) also depends on the tree’s species growth strategy, either acquisitive (ACQ) or conservative (CONS; see Supp. Table S1 and Fig. S1). Dots indicate trait values averaged at the leaf level, predicted from significant effects of the respective model. Notation in bold on top left of each panel indicates the significant terms (I: soil inoculation with species’ native microbiota; P: phosphorus fertilization; S: species growth strategy) with significant levels indicated as < 0.001 = ***, < 0.01 = **, < 0.05 = * (see Table 1). Error bars represent two standard errors around the mean. Leaf Mg (b) and leaf K (**e**) values were log-transformed for the analysis and back-transformed for illustration purpose.

While the relative species classification into acquisitive and conservative ones was based on all nine leaf traits, it was also reflected in single trait values, with for example leaf P and leaf N (Fig. S2a and b) having higher values in trees belonging to acquisitive species, and oppositely for leaf C:N (Fig. S2c; Table 1). As for the effect of the soil treatments on trait values, it was predominantly the traits related to an acquisitive growth strategy which responded. Soil inoculation increased leaf P, leaf Mg (Fig. 2a and b; Table 1) and leaf K, however, for the latter only when P was added as well (Fig. 2e). The addition of P lowered both acquisitive- and conservative-related trait values, specifically leaf P, leaf K (only on sterile soil), and leaf C for trees from conservative species (Fig. 2c,d and e).

**Table 1:** Mixed-effects models (anova, type III sum of squares) for effects of Diversity (i.e., monospecific or heterospecific tree species pair), Ino. (soil inoculation with species’ native microbiota), P fert. (phosphorus fertilization), Strategy (species growth strategy, see Table S1 and Fig S1) and their interaction on the different leaf traits.

Leaf trait values	Predictor	NumDf	DenDF	F-value	*p*-value
SLA	Diversity	1	192.53	4.66	**0.032**
	Strategy	1	7.36	10.23	**0.014**
	Diversity * Strategy	1	237.85	7.36	**0.007**
C:N	Strategy	1	6.55	14.85	**0.007**
C	Diversity	1	11.71	0.59	0.459
	Strategy	1	7.55	0.11	0.747
	P fert	1	181.82	15.23	**< 0.001**
	Diversity * P fert	1	175.24	5.58	**0.019**
	Strategy * P fert	1	291.44	8.56	**0.004**
N	Strategy	1	7.05	25.57	**0.001**
Mg	Diversity	1	14.25	1.00	0.335
	P fert	1	178.41	0.72	0.396
	Ino	1	179.39	4.23	**0.041**
	Diversity * P fert	1	177.91	6.40	**0.012**
Ca	Diversity	1	191.00	2.45	0.119
	Ino	1	190.98	2.90	0.090
	P fert	1	190.83	0.94	0.333
	Diversity * Ino	1	190.82	0.00	0.959
	Diversity * P fert	1	190.74	4.36	**0.038**
	Ino. * P fert	1	191.30	0.08	0.776
	Diversity * Ino. * P fert	1	191.13	4.28	**0.040**
K	Ino	1	180.83	1.08	0.299
	Diversity	1	9.66	7.65	**0.021**
	Strategy	1	6.89	0.02	0.903
	P fert	1	176.04	1.06	0.305
	Diversity * Strategy	1	11.87	0.99	0.340
	Ino. * Diversity	1	179.98	2.46	0.119
	Ino. * Strategy	1	239.79	0.00	0.952
	Ino. * P fert	1	177.08	4.24	**0.041**
	Ino. * Diversity * Strategy	1	237.40	3.94	**0.048**
P	Ino	1	186.50	3.93	**0.049**
	P fert	1	185.42	14.15	**< 0.001**
	Strategy	1	6.51	12.58	**0.011**

Leaf trait values for C:N, Mg, Ca and K are log-transformed.

Significant effects at the 0.05 level are indicated in bold.

**Figure 3:**
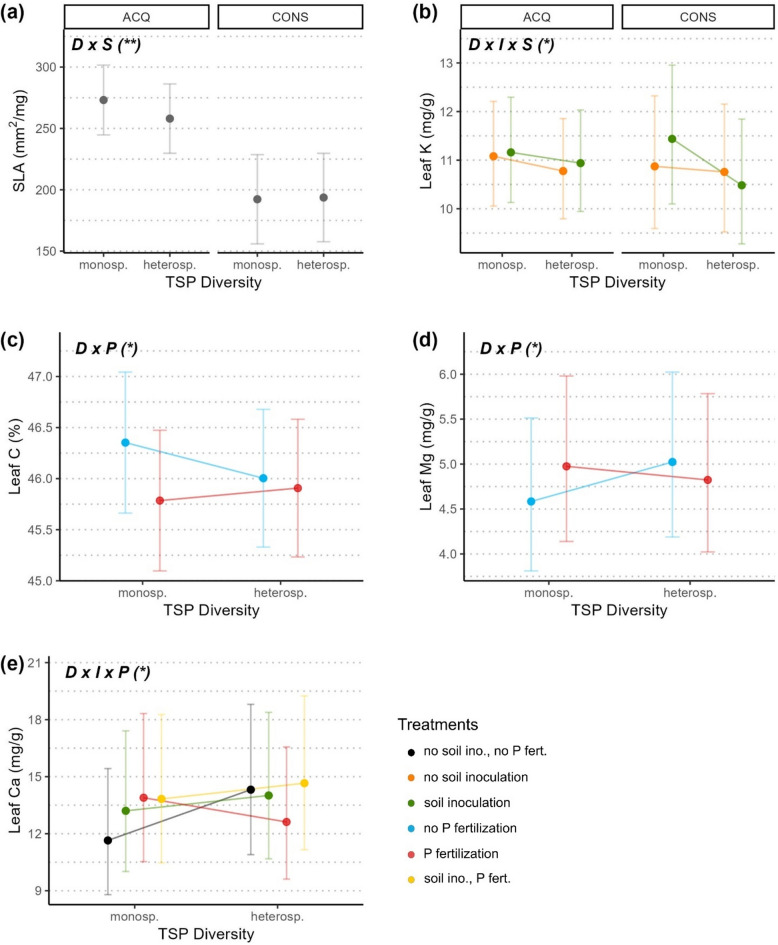
Effect of the tree species pair diversity (mono- or heterospecific TSP; (**a**) and its interaction with soil inoculation (**b**), phosphorus fertilization (**c**, **d**) and both soil treatments (**e**) on leaf traits. Effects of diversity (**a**) and diversity’s interaction with soil inoculum (**b**) on SLA and leaf K respectively also depends on the tree’s species growth strategy, either acquisitive (ACQ) or conservative (CONS; see Supp. Table S1 and Fig. S1). Dots indicate trait values averaged at the leaf level predicted from significant effects of the respective model. Notation in bold on top left of each panel indicates the significant terms (D: tree species pair diversity; I: soil inoculation with species’ native microbiota; P: phosphorus fertilization; S: species growth strategy) with significant levels indicated as < 0.001 = ***, < 0.01 = **, < 0.05 = * (see Table 1). Error bars represent two standard errors around the mean. Leaf K (**b**), leaf Mg (**d**) and leaf Ca (**e**) values were log-transformed for the analysis and back-transformed for illustration purpose.

Regarding the effects of diversity, trees belonging to monospecific TSPs displayed a higher SLA than heterospecifics, but only for trees from acquisitive species (Fig. 3a; Table 1). For leaf K, increasing diversity (from monospecific to heterospecific TSPs) had a negative effect on trait values in both inoculated and sterile soil, and for both species growth strategies. Soil inoculation seemed however to amplify this negative effect in trees from conservative species (Fig. 3b).

The addition of P yielded opposite results for conservative- (leaf C; Fig. 3c) and acquisitive-related traits (leaf Mg; Fig. 3d) regarding the effect of diversity. Without added P, leaf C tended to decrease with increasing diversity, and inversely to increase with diversity when P was added. We found the opposite for leaf Mg, which tended to decrease with increasing diversity when P was added, but to increase with increasing diversity without P addition. While trees in heterospecific TSPs seemed not or only slightly negatively affected by P addition for both traits, trees in monospecific TSPs showed a strong decrease in leaf C, but an apparent increase in leaf Mg with added P.

Without soil treatments, the increase in diversity had a positive effect on leaf Ca (Fig. 3e). Soil inoculation (both with and without P addition) seemed to buffer this positive effect of diversity. Contrarily, P addition without soil inoculation tended to reverse the diversity effect, and leaf Ca decreased in heterospecific TSPs when only P was added. Compared to the baseline situation (no addition of P or soil inoculation; black line), leaf Ca was higher for trees in monospecific TSPs but lower in heterospecific ones when only one of the soil treatments was applied. The joint effect of soil treatments resulted in the highest values of Ca for trees in both monospecific and heterospecific TSPs, also increasing with increasing diversity.

### Within-tree trait variation

**Figure 4:**
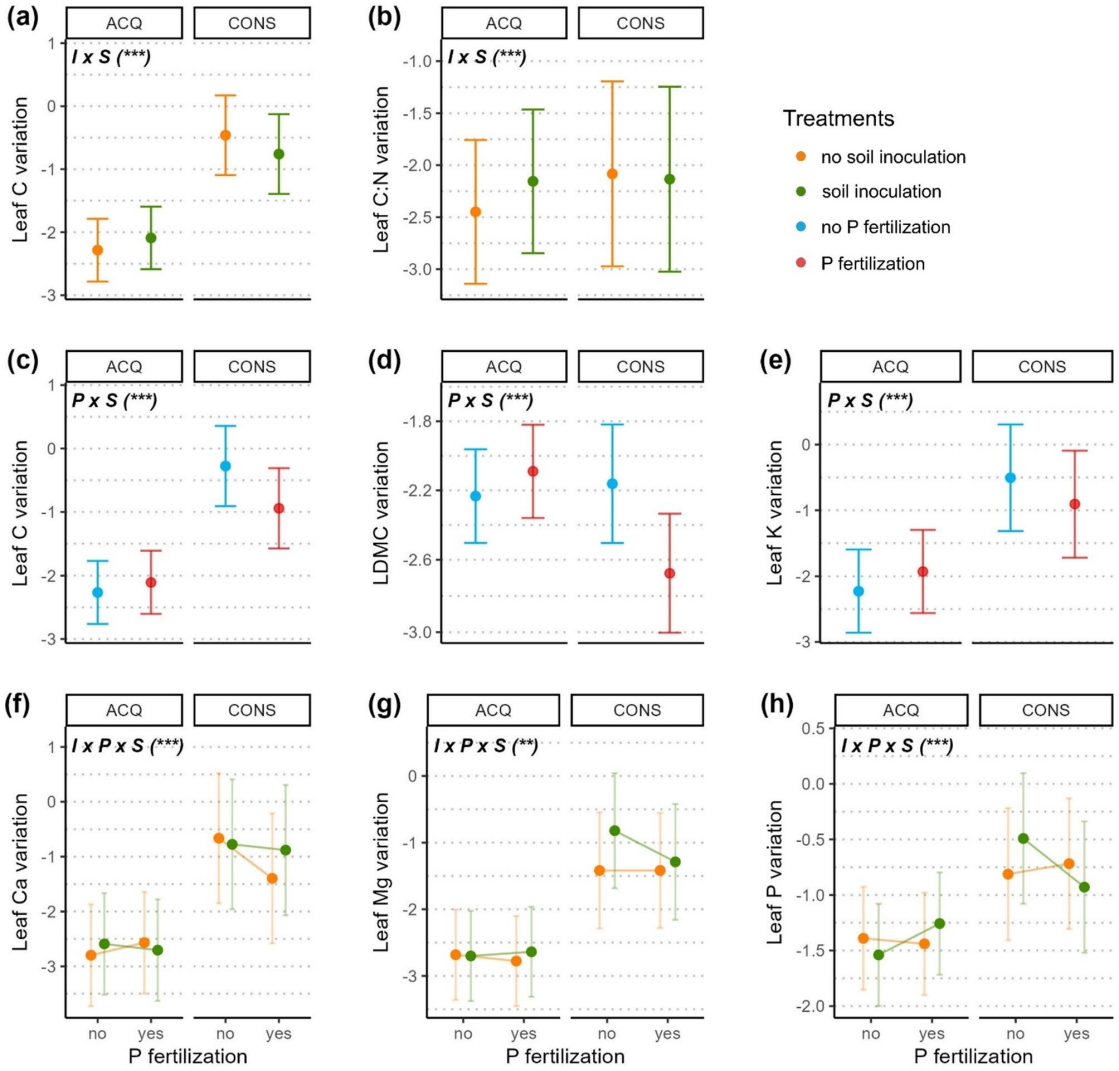
Effect of soil inoculation (**a**, **b**), phosphorus fertilization (**c**, **d**, **e**) and the interaction of both soil treatments (**f**, **g**, **h**) on leaf trait variation. All effects also depend on the tree’s species growth strategy, either acquisitive (ACQ) or conservative (CONS; see Supp. Table S1 and Fig. S1). Dots indicate trait variation within each individual, calculated as log-transformed within-tree Rao’s Q, predicted from significant effects of the respective model. Notation in bold on top left of each panel indicates the significant terms (I: soil inoculation with species’ native microbiota; P: phosphorus fertilization; S: species growth strategy) with significant levels indicated as < 0.001 = ***, < 0.01 = **, < 0.05 = * (see Table 2). Error bars represent two standard errors around the mean.

**Figure 5:**
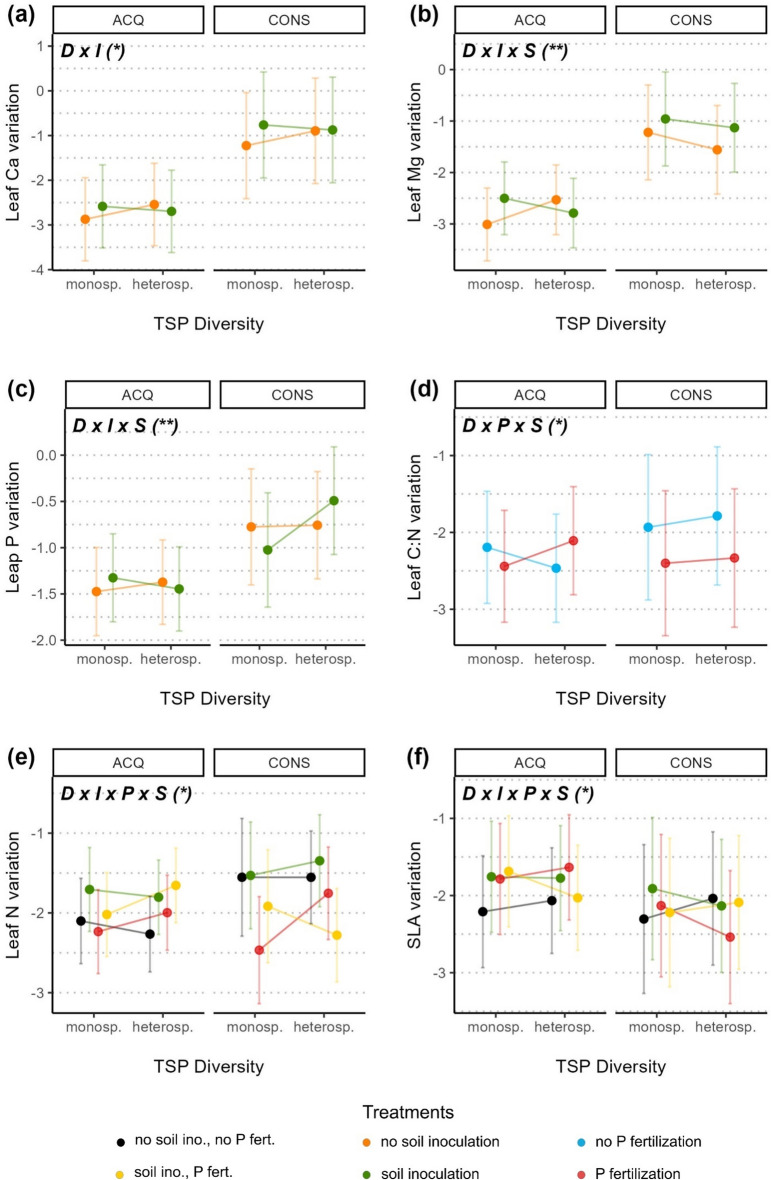
Effect of the tree species pair diversity (mono- or heterospecific TSP) interaction with soil inoculation (**a**, **b**, **c**), phosphorus fertilization (**d**) and both soil treatments (**e**, **f**) on leaf trait variation. All effects also depend on the tree’s species growth strategy, either acquisitive (ACQ) or conservative (CONS; see Supp. Table S1 and Fig. S1). Dots indicate trait variation within each individual, calculated as log-transformed within-tree Rao’s Q, predicted from significant effects of the respective model. Notation in bold on top left of each panel indicates the significant terms (D: tree species pair diversity; I: soil inoculation with species’ native microbiota; P: phosphorus fertilization; S: species growth strategy) with significant levels indicated as < 0.001 = ***, < 0.01 = **, < 0.05 = * (see Table 2). Error bars represent two standard errors around the mean.

Overall, the responses observed for trait variation were less consistent than those of trait values. The interaction of soil treatments (P addition and soil inoculation) as well as the interaction of soil treatments with species diversity mostly affected the variation of traits related to an acquisitive growth strategy, but not of traits related to a conservative strategy (Figs. 4, 5; Table 2). For the latter, the direction of treatment effects on trait variation mostly depended on the species’ growth strategy (Fig. 4).

**Table 2:** Mixed-effects models (anova, type III sum of squares) for effects of Diversity (i.e., monospecific or heterospecific tree species pair), Ino. (soil inoculation with species’ native microbiota), P fert. (phosphorus fertilization), Strategy (species growth strategy, see Table S1 and Fig S1) and their interaction on the different leaf traits within-tree variation.

Within-tree trait variation	Predictor	NumDf	DenDF	F-value	p-value
SLA	Diversity	1	15.17	0.10	0.759
	Ino	1	168.44	2.56	0.112
	P fert	1	167.93	0.01	0.911
	Strategy	1	6.76	0.32	0.590
	Diversity * Ino	1	167.36	0.78	0.379
	Diversity * P fert	1	167.27	0.85	0.357
	Ino. * P fert	1	168.87	2.00	0.160
	Diversity * Strategy	1	15.43	0.04	0.847
	Ino. * Strategy	1	201.79	0.14	0.713
	P fert. * Strategy	1	201.12	4.94	**0.027**
	Diversity * Ino. * P fert	1	169.13	1.02	0.315
	Diversity * Ino. * Strategy	1	200.74	1.54	0.216
	Diversity * P fert. * Strategy	1	200.66	0.00	0.996
	Ino. * P fert. * Strategy	1	203.23	3.75	0.054
	Diversity * Ino. * P fert. * Strategy	1	203.36	5.72	**0.018**
LDMC	P fert	1	200.59	5.97	**0.015**
	Strategy	1	6.80	1.49	0.263
	P fert. * Strategy	1	2137.33	64.08	**< 0.001**
C:N	Diversity	1	15.09	0.28	0.602
	P fert	1	177.20	6.52	**0.012**
	Ino	1	181.66	1.97	0.162
	Strategy	1	6.46	0.11	0.746
	Diversity * P fert	1	175.05	2.20	0.140
	Diversity * Strategy	1	15.22	0.11	0.743
	P fert. * Strategy	1	216.29	15.17	**< 0.001**
	Ino. * Strategy	1	1970.39	13.58	**< 0.001**
	Diversity * P fert. * Strategy	1	215.50	5.59	**0.019**
C	Ino	1	184.57	0.26	0.610
	P fert	1	183.27	6.09	**0.015**
	Strategy	1	6.81	15.84	**0.006**
	Ino. * Strategy	1	2907.98	36.07	**< 0.001**
	P fert. * Strategy	1	2904.05	101.94	**< 0.001**
N	Diversity	1	15.29	1.04	0.323
	Ino	1	174.77	4.78	**0.030**
	P fert	1	174.41	10.41	**0.001**
	Strategy	1	6.90	0.26	0.627
	Diversity * Ino	1	172.82	0.83	0.363
	Diversity * P fert	1	172.95	1.83	0.178
	Ino. * P fert	1	177.01	0.44	0.508
	Diversity * Strategy	1	15.45	0.06	0.804
	Ino. * Strategy	1	202.85	3.52	0.062
	P fert. * Strategy	1	202.38	15.18	**< 0.001**
	Diversity * Ino. * P fert	1	177.08	2.46	0.118
	Diversity * Ino. * Strategy	1	202.18	3.10	0.080
	Diversity * P fert. * Strategy	1	202.01	1.28	0.260
	Ino. * P fert. * Strategy	1	208.14	0.02	0.877
	Diversity * Ino. * P fert. * Strategy	1	208.08	4.55	**0.034**
Mg	Diversity	1	15.56	0.33	0.575
	P fert	1	179.31	1.91	0.168
	Ino	1	176.64	6.43	**0.012**
	Strategy	1	6.80	7.54	**0.030**
	Diversity * Ino	1	174.00	2.61	0.108
	P fert. * Ino	1	179.39	0.75	0.388
	Diversity * Strategy	1	15.79	2.02	0.175
	P fert. * Strategy	1	2278.70	5.81	**0.016**
	Ino. * Strategy	1	209.89	2.21	0.139
	Diversity * Ino. * Strategy	1	207.37	9.47	**0.002**
	Ino. * P fert. * Strategy	1	2280.15	11.98	**0.001**
Ca	Ino	1	198.44	2.12	0.147
	Diversity	1	192.62	1.09	0.298
	P fert	1	197.74	3.00	0.085
	Strategy	1	6.16	5.36	0.059
	P fert. * Strategy	1	2719.88	25.81	**< 0.001**
	Diversity * Ino	1	192.48	4.46	**0.036**
	Ino. * P fert	1	197.39	0.46	0.499
	Ino. * Strategy	1	2734.55	3.19	0.074
	Ino. * P fert. * Strategy	1	2716.33	26.71	**< 0.001**
K	P fert	1	204.16	0.37	0.544
	Strategy	1	6.16	7.00	**0.037**
	P fert. * Strategy	1	2354.09	67.33	**< 0.001**
P	Diversity	1	183.94	2.55	0.112
	P fert	1	188.55	0.12	0.733
	Ino	1	184.54	0.08	0.774
	Strategy	1	6.34	3.15	0.124
	Diversity * Ino	1	184.74	0.77	0.380
	P fert. * Ino	1	188.80	0.38	0.536
	Diversity * Strategy	1	196.31	3.67	0.057
	P fert. * Strategy	1	2289.43	12.73	**< 0.001**
	Ino. * Strategy	1	221.29	0.02	0.877
	Diversity * Ino. * Strategy	1	221.29	7.43	**0.007**
	Ino. * P fert. * Strategy	1	2295.95	28.61	**< 0.001**

All trait variation, calculated as within-tree Rao’s Q values, were log-transformed.

Significant effects at the 0.05 level are indicated in bold.

Either the presence of inoculum (for leaf C and CN) or the addition of phosphorus (for leaf C, LDMC, leaf K) resulted in an overall increase of within-tree variation for individuals of acquisitive species, while we observed the opposite for conservative species (Fig. 4a–e).

When looking at the joint effect of the treatments, we found that for individuals belonging to acquisitive species, any combination of the treatments seemed to yield little effect, except for an increase of variation of leaf P when phosphorus was added to inoculated soil (Fig. 4h). In contrast, for conservative species, P addition to inoculated soil led to a decrease of variation of leaf Ca, Mg and P, while P addition to sterile soil led to mixed responses of these traits (Fig. 4f–h).

Increasing diversity was associated with an increase of variation in sterile soil, but a decrease of variation in inoculated soil for leaf Ca, Mg, and P (Fig. 5a,b and c). Conservative species were less consistent in this trend, as shown by variation of leaf Mg decreasing in sterile soil, and increasing for leaf P in inoculated soil. For trees in monospecific TSPs, variation of these traits was similar or higher in inoculated than in sterile soil (except for leaf P of conservative species). On the contrary, trees in heterospecific TSPs displayed similar or higher variation of leaf Ca, Mg and P in sterile than in inoculated soil, but this was only the case for acquisitive species.

Addition of P modified the effect of diversity on leaf C:N variation for individuals from acquisitive species, reversing a decrease in variation without P to an increase with P (Fig. 5d). Variation of acquisitive trees in monospecific TSPs tended to be lower with P addition than without, contrary to trees in heterospecific TSPs. For conservative species, the effect of increased diversity tended to increase variation of leaf C:N, independently from P addition. Moreover, the addition of P decreased variation of conservative TSPs, in particular for trees in heterospecific pairs.

For the variation of SLA and leaf N, we found positive and negative trends as well as no diversity effects, strongly depending on the combination of soil treatments and species’ strategies (acquisitive or conservative) and differing between the two traits (Fig. 5e and f). With no additional access to nutrients (no soil inoculation, no P fertilization), leaf N variation tended to decrease or showed no response with increasing diversity, but increased for SLA. For both traits, addition of nutrients resulted most often into higher variation in acquisitive species and lower variation in conservative ones (with P fertilization alone), while variation tended to be higher or unchanged on inoculated soil (soil inoculation alone). Together with increasing diversity, addition of P tended to increase variation (except for SLA of conservative species). Meanwhile, on inoculated soil, increasing diversity led to mixed results regardless of P addition.

## Discussion

In a controlled environment, we investigated species diversity effects on trait values and trait variation of individual trees, and how they are modulated by manipulated soil phosphorus and species’ native microbiota. Most notably, with a single exception, we did not detect any response to diversity which was independent of soil treatments, and often trait responses differed between acquisitive and conservative species. We observed trait shifts towards an overall tree acquisitiveness (according to H1) as well as a tendency for trait variation to increase (contrary to H2) with increasing diversity.

In the absence of phosphorus fertilization, increased species diversity partly increased tree acquisitiveness (leaf C, leaf Mg, leaf Ca), giving limited support for H1. These findings are consistent with the idea that diversity enhances resource availability through resource-use complementarity or facilitation^40,41^, which in turn fosters an acquisitive growth strategy^42^. In terms of trait variation within individual trees, we found little evidence supporting our second hypothesis that under low nutrient availability (here, without inoculation and P addition) beneficial effects of diversity reduce competition and thus the need for variation. On the contrary, increasing species diversity tended to increase intra-individual variation for trees from both acquisitive and conservative species. However, with a maximum of two species, interacting for a limited portion of their lifespan, we were not able to detect effects of diversity that would be more pronounced at a higher range of diversity values, and intensify over time.

The only diversity effect independent from soil conditions was a decrease of SLA for acquisitive species with increasing diversity, contrasting our expectations (H1). Considering that SLA is mainly driven by light^43^, this result likely reflects the spatially complementary crown architecture of trees in heterospecific pairs, compared to more similarly shaped crowns in monospecific ones. Assuming that acquisitive species grow faster than conservative ones, light availability for acquisitive species is higher in species mixtures, leading to lower SLA^44,45^. Additionally, the shift towards lower SLA values in response to an increasing diversity could be influenced by competitive advantages of fast-growing species at early stages of growth^46^. It is likely that in our experiment, with trees being only one year old, species identities were strongly expressed and fast-growing species tended to dominate the competitive interaction. Given the typically strong association between SLA and leaf K, it is not surprising here that increased diversity also reduced leaf K (again in contrast to our hypothesis). The observed decrease of leaf K for conservative species on inoculated soil goes in line with the assumption that conservative species have a disadvantage in species mixtures, that is reinforced under enhanced nutrient availability through soil inoculation.

A lack of phosphorus availability is known to strongly limit plant growth, in particular in tropical and subtropical forests^47,48^. As a limiting nutrient, adding phosphorus not only lifts this limitation but also enables the uptake of other nutrients^49^. In our study, in species mixtures and under P fertilization, we observed lower values for traits related to both an acquisitive (leaf Mg, leaf Ca) or conservative (leaf C) growth strategy, as well as a decrease in trait values with increasing diversity (leaf Mg, leaf Ca). This suggests a greater tree biomass in response to more available nutrients, resulting in lower amounts of nutrients per mass unit of leaf material. This so called effect of biomass dilution^50^ is also reflected in lower values of leaf P and K in the P-addition treatment. Hence, we can conclude, aligning with our first hypothesis, that P fertilization fosters an investment of resources in faster growth rather than long-lasting structures, which also led to lower values in traits associated with a conservative growth strategy, such as leaf C. Overall, these findings suggest that biomass dilution can be enhanced in more diverse settings.

However, we also observed notable exceptions to this pattern, such as an increase in leaf C with increasing diversity and P fertilization, and higher values for leaf Mg and in leaf Ca in monospecific tree pairs with P fertilization. These mixed results reflect the variety of responses found in other studies investigating the effect of P fertilization, which have been found to be highly species specific^51–53^, and may obscure a strengthening of positive species diversity effects. For example, in controlled experiments the ability to benefit from an addition of P has been shown to be higher for species already growing in P-rich soils, while species adapted to low-P soils performed better under low-P conditions^54–56^. This highlights the importance of considering P availability together with the variety of species’ P-use efficiencies, instead of focusing only on the number of plant species as one measure of biodiversity. Including such processes might also improve the translation of findings from controlled experiments to natural forest communities^57^.

Supporting our second hypothesis, with P fertilization, diversity led to an increase of variation of leaf C:N, leaf N and SLA (however, not completely independent from soil inoculation and species growth strategy). These results expand on conclusions of previous studies, which reported at a coarser scale that increased resource availability and hence a more favourable environment enables higher trait variation^15,58^. When adding phosphorus, we found higher variation of leaf C:N, leaf N and SLA in heterospecific tree pairs and lower variation in monospecific ones for trees from acquisitive species (except for SLA), and inversely for heterospecific trees from conservative species. It is likely that because of strong competition within monospecific pairs of acquisitive species and the associated necessity to vary for mitigating competition, the addition of resources reduces the need for variation by providing a more favourable environment to the competitors. Inversely, conservative species within heterospecific pairs can use additional resources to optimize their variation ^27^. Taken together, the effects of P fertilization associated to diversity indicated synergistic effects on trait variation.

As expected in H1, inoculated soil seemed to increase resource availability and promoted an acquisitive growth strategy, reflected by higher values of leaf P, leaf Mg and leaf K (the latter only under P addition). However, while traits also shifted towards higher acquisitiveness following soil inoculation in monocultures (leaf K, leaf Ca), this was not consistent in species mixtures. Our assumption was that soil inoculation provides fungi and bacteria creating positive association with plants’ roots (through mycorrhiza, enhanced decomposition or nutrient cycling)^34,59^. In our experiment, it seems that at this young stage individuals of the same species growing together solicited more efficiently the microbiota specifically interacting in a positive way with this species. However, the same microbiota may be detrimental to a different species and could hinder plant resource acquisition and growth, leading to more conservative trait values. For example, soil biota can compete with plants for the same resources^60^, or contain pathogens detrimental to plants or their symbionts^61^, which might explain the observed negative or inconsistent effects of soil inoculation in species mixtures. Hence, our findings contrast other studies that found microbiota’s effect at high species diversity to be beneficial to plant growth, for example in diluting a species own detrimental biota^62^, enhancing complementarity in resource-use (through microbe mediated resource partitioning)^63^, or increasing microbial activity^64^. One might argue that we simply did not reach a level of diversity high enough to witness positive interactions between microbiota and species diversity, but it might also point to the fact that the role of microbiota switches during life stages^65^. Indeed, it is possible that we observed the effects of microbiota in the specific context of young conspecific individuals for which it is beneficial to support each other for establishing a stable population, while the negative role of intraspecific competition, that was expected as a baseline for our hypotheses, increases only at later stages. Hence, our results are describing an interaction particular to this early life stage, which might not be reflected over trees’ long lifespan.

Similarly as for its effect on trait values, microbiota’s interaction with diversity did not follow our hypothesis regarding trait variation. While we observed the expected higher trait variation through soil inoculation (H2) mainly in monospecific tree pairs (leaf Ca, leaf Mg, leaf P, leaf N, SLA), trait variation mostly decreased with diversity under inoculated conditions (leaf Ca, leaf Mg, leaf P, SLA).

Overall, we could not identify consistent positive interactions between the two soil treatments. Despite similar effects on traits when considered independently, P fertilization and soil inoculation did not show any synergy, and their respective interaction with increased diversity led to opposite patterns. Consequently, the effect of both soil treatments with increased diversity was equally inconsistent. These results reinforce the idea of different roles of soil nutrients and microbiota in driving plant growth.

When considering the trees’ responses to diversity and soil treatments, we observed substantial differences between acquisitive and conservative species especially in their variation, but partly also in their trait expression. This suggests that while the modified conditions enabled flexibility in the individuals’ responses, the direction of the individual trait shifts and the amount of within-individual trait variation were not strongly constrained by the species’ growth strategy. In addition, independently from the tree species’ growth strategy, the traits related to an acquisitive growth strategy were the most responsive to the diversity and soil treatments for both trait values and variation. Hence, our results indicate that assessing the potential of trait variation and associated growth strategies at an individual level could be preferable when aiming at understanding how local effects of diversity alter plant resource-use and adaptations to changing environmental conditions. However, because of its focus on local interactions, our study uses naturally co-occurring species whose acquisitiveness is relative to the set of species we consider, with difference between life strategies less stark than as in the initially described leaf economics spectrum. These aspects add to the challenge of bridging the gap between local and global scale when investigating mechanisms behind species interactions.

## Conclusion

In this study, we make a first step towards disentangling how soil conditions alter diversity effects on both trait values and trait variation of individual young trees. Our results highlight that the effects of diversity clearly depend on soil conditions. While phosphorus fertilization seemed to consolidate positive effects of diversity on tree acquisitiveness and enhance their variation, the presence or absence of the species’ native microbial community yielded unexpected responses, likely inherent to the complexity of its composition and functioning. Individuals with a relatively fast-growing strategy seemed to benefit more from improved soil conditions and diversity. Hence, traits and trait combinations should be considered in the light of plants’ growth strategies and their interactions when investigating species diversity effects. We encourage future studies to continue towards capturing a more holistic understanding of these interactions, by considering not only leaf traits, but also belowground as well as wood traits and their associated spectrum, together with a wider selection of species, representative of a broader spectrum of life strategies. Taken together, our findings demonstrate the dependency of plant interactions to their local growth conditions at an early life stage, suggesting a high level of individual plants’ adaptability in response to a changing biotic and abiotic environment.

## Methods

We conducted an 18-month experiment under controlled conditions in an experimental greenhouse located in the botanical garden of Halle, Germany.

### Species combinations

Understanding tree interactions is essential to determine the role of local processes in driving the BEF relationship^66^. To complement and deepen findings from the field, investigated in the biodiversity experiment BEF-China^20,27,64^, eight native species from subtropical China were selected from the species pool of the BEF-China experiment^67^ (see Supp. Table S1). While being long-lived trees species, all species were observed in the field to be growing relatively fast, and were shown to have contrasted leaf traits at later life stage (in nine year old trees)^25^ and thus suitable for the duration of the controlled experiment.

Seeds were collected in the Qianjiangyuan National Park in autumn 2018 (Zhejiang province, southeast China) or bought from a local seed supplier. The seeds were kept cool and dark over the winter months. In spring 2019, the seeds were germinated in germination trays filled with a sterilized 3:2 mixture of compost soil and sand in the glasshouse of the botanical garden in Halle (Saale), Germany. The seedlings stayed in the trays for 9 to 18 weeks and were then planted in July 2019 in ca. 30 L tubes (20 cm diameter, 100 cm height) filled with a sterilised 1:1 mixture of soil and sand. At this point, individuals measured on average between 2.1 and 10.1 cm depending on the species. The soil used represents brown earth and was collected from the mineral layer (A-horizon, up to a depth of ca. 30 cm) of forests close to the city of Halle, Germany. At these sites, loess is located over sandy clay and sandy marl. We chose the German sites for collection because of their acidic (pH_KCl_ = 4.24) and comparably nutrient poor characteristics (C:N = 13 g/g; CEC_eff_ = 30 μmol_c_/g), which were similar to soil conditions in the BEF-China experimental site (pH_KCl_ = 3.8; C:N = 11 g/g; CEC_eff_ = 56 μmol_c_/g; see also^68^). The soils at Site A of the BEF-China experimental platform have previously been described as Cambisols, together with Regosols on ridges and crests, Acrisols on slopes and Gleysols and Anthrosols in foot slopes and valleys^68^. The German forest soil was sieved to 5 mm and mixed with washed sand (‘Sand’ according to the German Industrial Norm (DIN) 18196, with a maximum of 5% silt and sieved to 2 mm) from a commercial supplier before being sterilized with a soil steam sterilizer (active steaming for 20 min with 200–250 °C hot steam). After sterilization the pH was 5.8 in the soil:sand mixture.

The eight species were separated in two sets of four (Supp. Table S1). Two individuals were planted per tube, including all possible species combinations within each set of four species, totalling 20 different combinations (six heterospecific and 4 monospecific tree species pairs for each set, referred to as TSPs).

### Soil treatments

In each tube, the upper 5% of the tube soil volume consisted of a mixture of the sterilized background substrate with soil from the native region of the studied species (China). This native soil was collected (up to a depth of 20 cm) in the species’ monocultures at the BEF-China main experiment, site A^67,68^ and blended. The mixture of native soil was used either with its microbiota kept alive as an inoculum (+ Ino), or sterilised, to provide a control (− Ino; Fig. 1c). The tubes where then covered with a 2 cm layer of sand to prevent external pathogens.

After planting, each species combination received every three weeks 15 ml of a fertilizer solution consisting of nitrogen, calcium, magnesium, potassium and either phosphorus (+ P) or water (− P; Fig. 1c). The phosphorus addition corresponded to an annual amount of 10 kg P/ha.

We applied a full factorial design with all possible combinations of soil treatments and diversity levels (mono- or heterospecific TSPs), resulting in eight different treatment combinations (Fig. 1c). The 10 different species combinations per species set (4 monocultures and 6 heterospecific mixtures) led to 40 different tubes, which were replicated three times (overall 120 tubes). The replicates were evenly distributed across three cabins in the greenhouse (later used as ‘block’ effect in the statistical analysis). Together with the second species set, the total number of tubes amounted to 240, distributed across 6 greenhouse cabins. The greenhouse cabins were kept in subtropical conditions with 70–80% relative air humidity and 25/20 °C during the summer months and 15/10 °C over the winter. Water was individually provided to the tubes. Since the water requirements and consumption rates of the different species, but also those of different species combinations varied significantly, soil moisture was controlled for each tube individually at least once per day during the growing season and at least every second day during winter. Because of these individual requirements, it was not possible (and not meaningful) to provide the same amount of water to all tubes. Rather, the amount of water depended on the actual moisture of the tube’s soil, which was assessed visually and haptically by experienced staff of the botanical garden. By deciding for such an individual treatment of the tubes, we made sure that the plants were neither exposed to waterlogging nor drought conditions, which might have influenced our results in an unwanted way.

### Data collection

After more than one year of growth, trees were on average between 14.2 and 201.5 cm depending on the species. In August/September 2020, we collected for each tree between two and eight leaves equally spaced along the crown, with a leaf number adapted to the crown size, to ensure that this sample would be representative of the variation within the crown vertical spread. Leaves were collected on the side where two trees of a pair were the closest. Leaves were then immediately measured with an ASD FieldSpec4 Wide Resolution Field Spectroradiometer (Malvern Panalytical Ltd., Malvern, United Kingdom) to acquire leaf reflectance spectral data, over a 350 to 2 500 nm wavelength range^69^. A white diffuse reflectance target (Spectralon, Labsphere, Durham, New Hampshire, USA) was used as reference on which the device was regularly calibrated in parallel to the measurements. To minimize measurement errors, each individual spectral measurement was repeated three times for each leaf.

### Data processing

To predict trait values from leaf spectra, we used for each trait an existing partial least square regression model, which linked spectral data and laboratory-measured trait values for the same species as used here. These data were collected in the BEF-China Site A (Jiangxi, China) during August to October 2018^25^. With these trait-specific prediction models, a trait value was calculated for each repeated spectral measurement of each leaf. The predicted traits, reflecting the plants investment in growth and survival, were related to an acquisitive growth strategy, that is, involved for example in light acquisition and photosynthesis (SLA, leaf N), as well as respiration, nutrition, or chemical defence (leaf Mg, leaf K, leaf Ca, leaf P), or related to a conservative growth strategy, important for example for structural defence and integrity (LDMC, leaf C, leaf C:N)^70,71^. Prediction accuracy ranged from R2 = 24.2% to R2 = 88.9%^25^.

### Statistical analysis

Outlying predicted trait values were excluded for each trait on the base of a 99% confidence interval applied species-wise. We also excluded negative values and values with an outstanding standard error (exceeding five time the mean standard error). Trait values from the repeated measurements were then averaged, resulting in one value per leaf. Individuals with less than two sampled leaves, as well as individuals with no neighbour in the same tube (incomplete pairs) were excluded from the analyses, totalling between 404 and 412 trees (3030 and 3198 leaves), depending on the trait. We used the predicted trait values for two separate analyses on trait values and trait variation, respectively, as described below.

#### Species classification

As species’ growth strategies might affect their response to diversity, we grouped species accordingly. To do so, we performed a principal component analysis with each species’ traits averaged for each treatment combination. We then used the two resulting clusters as proxy for acquisitive and conservative species (Supp. Fig. S1, Table S1). This approach allowed us to conserve the general purpose of traits to characterise species interactions, but prevents us from describing species-specific behaviour.

#### Leaves’ trait values

We used the trait values averaged at the leaf level as response variable in linear mixed models for each of the nine studied traits. Leaf level trait values were explained by the species diversity of the TSP (i.e., Div, either monospecific or heterospecific TSP), the presence of soil inoculum instead of sterilized soil (Ino), fertilization with phosphorus (P), and the growth strategy of the species to which the tree belonged (i.e., Strategy, either acquisitive or conservative), and all their interactions. The species identity, species combination, as well as the tree identifier nested in the growing tube identifier, itself nested in the greenhouse chamber’s identifier, were added as crossed random factors (Table 1). To correct for heteroscedasticity and non-normality of the residuals, trait values were log-transformed for four out of nine traits (leaf C:N, leaf Mg, leaf Ca and leaf K).

#### Within-tree trait variation

In parallel, the leaf-level trait values of each individual tree were used to calculate Rao’s quadratic entropy (Rao’s Q) as a measure of within-tree trait variation. With setting weights and abundance to one, as all leaves within each tree were considered equal, we used the FD package to determine Rao’s Q for each trait for each tree, that is, the mean Euclidian distance between trait values of all sampled leaves within an individual.

We then fitted a linear mixed model for each of the nine studied traits, with all trees’ Rao’s Q explained by the same factors as for the trait values models above (species diversity of the TSP, presence of soil inoculum, fertilization with phosphorus, species growth strategy and all their interactions). The random structure was also the same as for trait values models, except for the absence of the tree identifier. To fulfil linear model requirements, Rao’s Q of all traits was log-transformed to correct for heteroscedasticity and non-normality of the model residuals.

The full models of both trait variation and trait values were simplified by stepwise removal of model terms based on significance at *p* < 0.05. *P*-values were extracted from F-statistics of type III sum of squares with Satterthwaite approximation for estimating the denominator degrees of freedom (Tables 1 and 2).

All statistical analyses were performed in R, version 4.0.4.

### Supplementary Information

Supplementary Information.

## Data Availability

The datasets generated and analysed during the current study are available from the corresponding author on reasonable request.
