# Supplementary material for: Soil conditions modify species diversity effects on tree functional trait expression
Source: Sci Rep. 2024 Jul 24;14:17114. doi: 10.1038/s41598-024-67512-w (PMC11269567; doi:10.1038/s41598-024-67512-w)
Supplement: Supplementary file 1 — Supplementary Information. [file 41598_2024_67512_MOESM1_ESM.pdf]

SUPPLEMENTARY MATERIAL

Davrinche A. & Haider S. Soil conditions modify species diversity effects on tree functional trait expression. *Scientific Reports.*

Table S1: List of tree species included in the study. Nomenclature according to The Flora of China (http://flora.huh.harvard.edu/china). Species were planted in pairs in all possible combinations within each set. Abb.: abbreviated name; Acq.: acquisitive; Cons.: conservative according to a PCA based on the measured functional traits (Fig. S1).

Species name	Abb.	Author	Family	Growth strategy	Set
Choerospondias axillaris	Ca	(Roxb.) B.L.Burtt & A.W.Hill	Anacardiaceae	Acq.	A
Koelreuteria bipinnata	Kb	Franch.	Sapindaceae	Acq.	A
Quercus fabri	Qf	Hance	Fagaceae	Acq.	B
Quercus serrata	Qs	Thunberg	Fagaceae	Acq.	A
Sapium sebiferum	Ss	(L.) Roxb.	Euphorbiaceae	Acq.	A
Cyclobalanopsis glauca	Cg	(Thunberg) Oersted	Fagaceae	Cons.	B
Rhus chinensis	Rc	Mill.	Anacardiaceae	Cons.	B
Schima superba	Ssu	Gardn. & Champion	Theaceae	Cons.	B

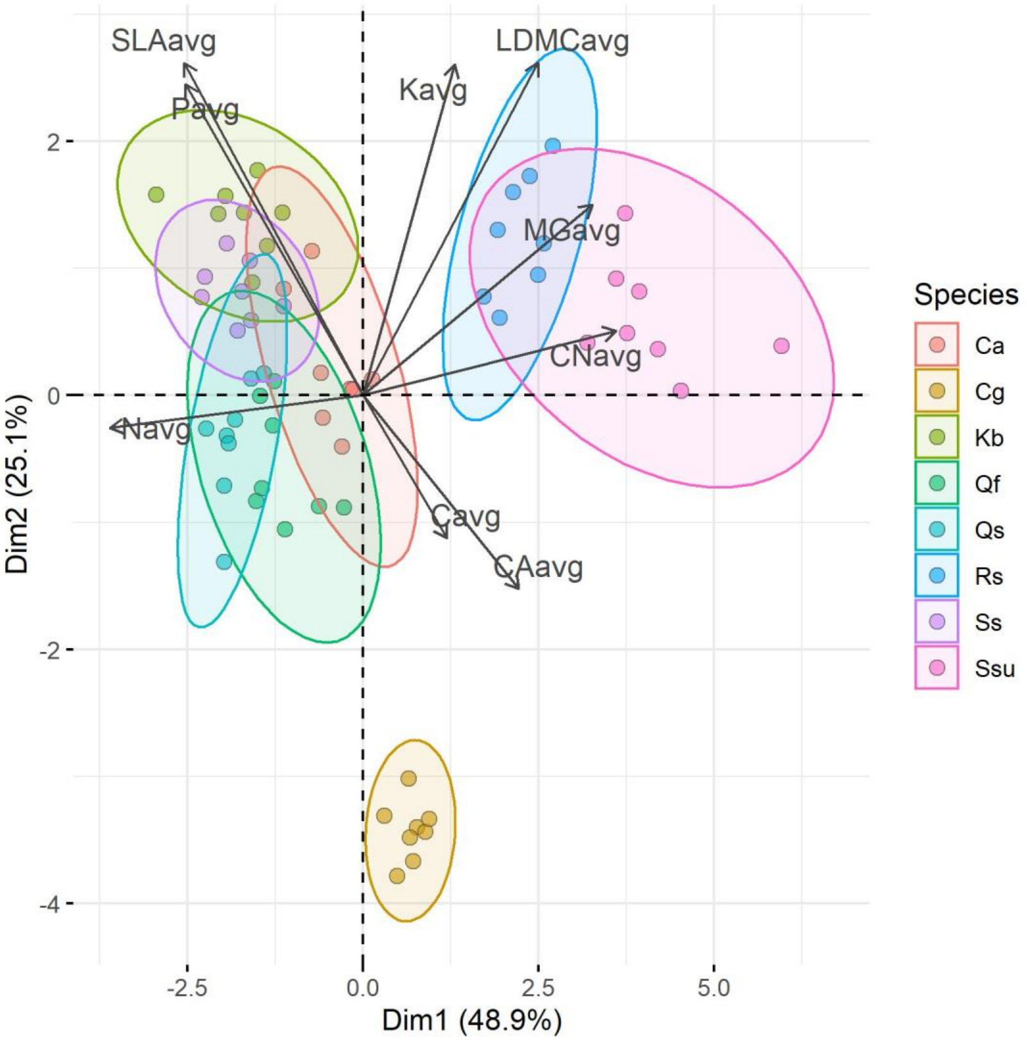

Fig. S1: Principal component analysis performed on all trait values, averaged for each treatment combinations of each species. The two groups delimited by the first dimension were used as separation between acquisitive (Ca, Kb, Qf, Qs and Ss) and conservative species (Cg, Rc, Ssu). Species abbreviations are detailed in Table S1.

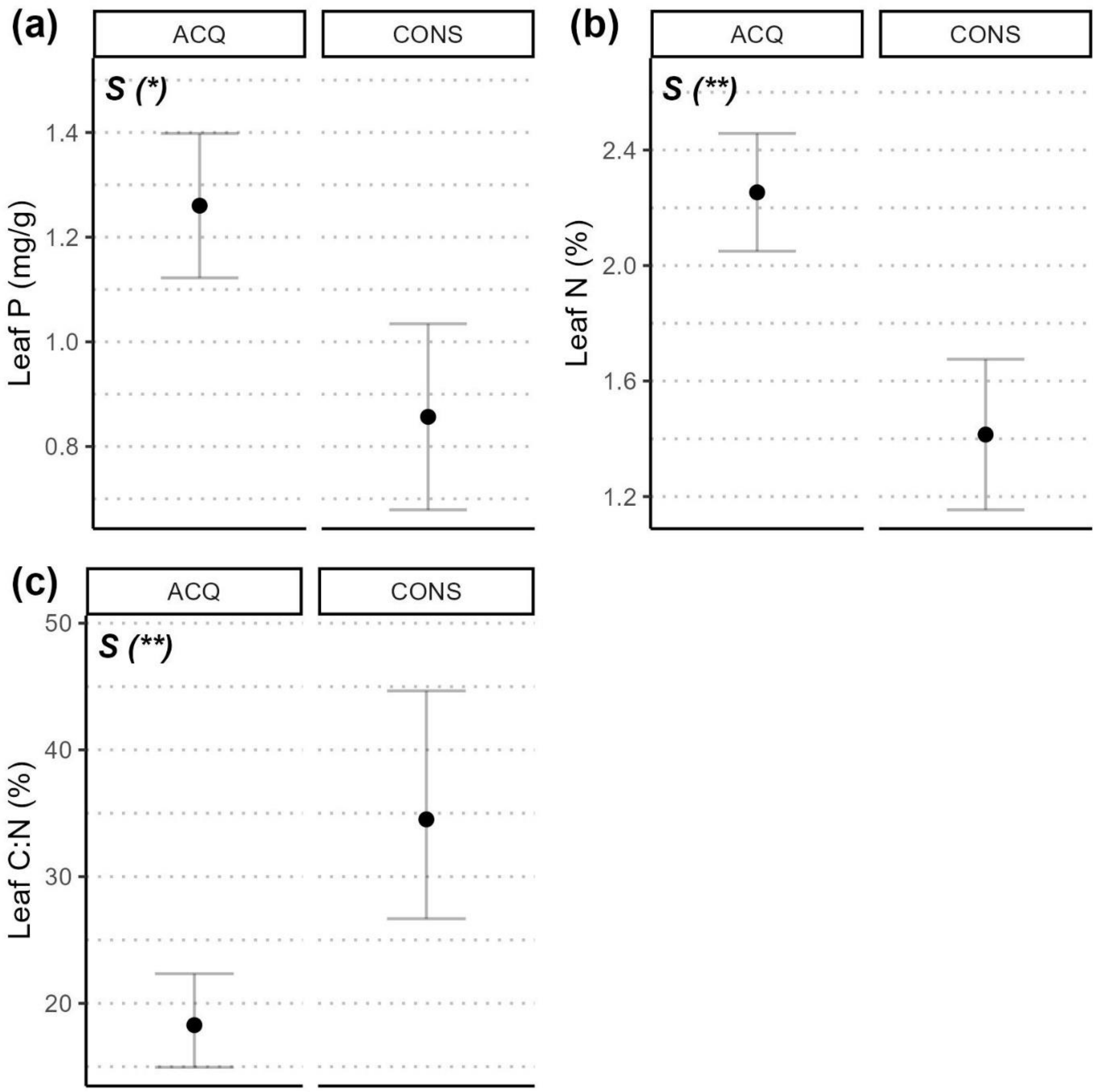

Fig. S2: Effect of trees species growth strategy on leaf traits (ACQ: acquisitive, CONS: conservative; see also Supp. Table S1 & Fig. S1). Dots indicate trait values averaged at the leaf level predicted from significant effects of the respective trait linear mixed model (see Table 1). Error bars represent two standard errors around the mean.
